# Development of a facile droplet-based single-cell isolation platform for cultivation and genomic analysis in microorganisms

**DOI:** 10.1038/srep41192

**Published:** 2017-01-23

**Authors:** Qiang Zhang, Tingting Wang, Qian Zhou, Peng Zhang, Yanhai Gong, Honglei Gou, Jian Xu, Bo Ma

**Affiliations:** 1 Single-Cell Center, CAS Key Laboratory of Biofuels and Shandong Key Laboratory of Energy Genetics, Qingdao Institute of Bioenergy and Bioprocess Technology, Chinese Academy of Sciences, Qingdao, 266101, China; 2 Key Laboratory for Sustainable Development of Marine Fisheries, Ministry of Agriculture, Yellow Sea Fisheries Research Institute, Chinese Academy of Fishery Sciences, Qingdao, 266071, China

## Abstract

Wider application of single-cell analysis has been limited by the lack of an easy-to-use and low-cost strategy for single-cell isolation that can be directly coupled to single-cell sequencing and single-cell cultivation, especially for small-size microbes. Herein, a facile droplet microfluidic platform was developed to dispense individual microbial cells into conventional standard containers for downstream analysis. Functional parts for cell encapsulation, droplet inspection and sorting, as well as a chip-to-tube capillary interface were integrated on one single chip with simple architecture, and control of the droplet sorting was achieved by a low-cost solenoid microvalve. Using microalgal and yeast cells as models, single-cell isolation success rate of over 90% and single-cell cultivation success rate of 80% were demonstrated. We further showed that the individual cells isolated can be used in high-quality DNA and RNA analyses at both gene-specific and whole-genome levels (*i.e.* real-time quantitative PCR and genome sequencing). The simplicity and reliability of the method should improve accessibility of single-cell analysis and facilitate its wider application in microbiology researches.

Single-cell analysis is attracting great interests in many frontiers of microbiological research, as single-cell imaging, isolation and sequencing techniques are providing the possibility to monitor phenotypic and genetic heterogeneity among isogenic populations during cell growth, stress resistance, metabolites accumulation and other bioprocesses^1^, and to select individual cells with desired properties for biotechnology applications^2^. On the other hand, as the majority of microbes on earth are yet to be cultured, single-cell isolation in combination with single-cell sequencing can help identification of unknown species from environmental samples or clinical specimens and investigation of microbial community structure and functions^3^.

Acquisition of an individual cell without hampering its bioactivity is usually the first and most key step in single-cell analysis, which includes separation of a cell from the bulk as well as delivery of this particular cell to downstream biological analyses. Compared with animal and plants cells, capture and moving of individual microbial cells can be much more difficult, due to their small size, irregular shape, spontaneous motility and relatively short life time. Therefore, development of approaches for high-efficient isolation of single microbial cells is always in requirement.

Serial dilution^4^ and micro-pipetting^5^ methods were used in early single-cell studies with the advantages of being cheap and easy to perform, however, they usually suffer greatly from being imprecise, hard to validate and prone to DNA contamination. More automated methods such as optical/magnetic tweezers^6^ Raman-activated cell sorting (RACS)^7^ and fluorescence-activated cell sorting (FACS)^8^ require expensive instruments that are equipped with laser beam, force clamp or fluorescence flow cytometer, which limits their wider applications.

Recently, microfluidics-based methodology has shown great potential in single-cell isolation with facile automation, accuracy and high efficiency^2^,^9^. Single-cell trapping systems based on on-chip valves and microchambers were demonstrated for individual environmental bacterial cells and combined with on-line digital PCR^10^ or whole genome amplification^11^,^12^. Moreover, a programmable droplet-based microfluidic reaction array formed by integrated pneumatic valves was developed for on-line real-time quantitative PCR (qPCR) and genomic DNA (gDNA) amplification of single *Escherichia coli* cells^13^. However, the intricate chip design and highly-integrated system considerably raised the barrier to entry in single-cell analysis. Thus a more convenient and flexible platform which is able to isolate single microbial cells with high efficiency, as well as to be integrated with conventional protocols and instrumentation for downstream analyses (i.e. quantitative PCR or genomic sequencing on single-cell level) is highly desired.

Here, we developed a facile droplet microfluidic device by integrating cell encapsulation, droplet inspection, single-cell droplet sorting and exporting on one chip. A unique flow controlling technique based on capillary-tuned solenoid microvalve suction effect developed in our previous study^14^ was shown to be capable of on-demand single-cell isolation. A robust interface between the chip and the collection tube was enabled via a capillary interface. All steps were realized by easy-to-use and low-cost technologies, which ensured the simplicity and thus accessibility of this platform. In microalgal and yeast cells, single-cell isolation success rate of over 90% was achieved, and the generated single-cell droplets were readily dispensed into conventional standard containers such as PCR tubes and 96-well plates. Furthermore, subsequent single-cell cultivation experiments suggested minimal interference of cell vitality by the isolation method, while DNA/RNA analyses of the isolated cells at both gene-specific and whole-genome levels demonstrated ability of the method to couple with downstream functional genomic analysis.

## Results and Discussion

### Design and operation of the microdevice

**Figure 1:**
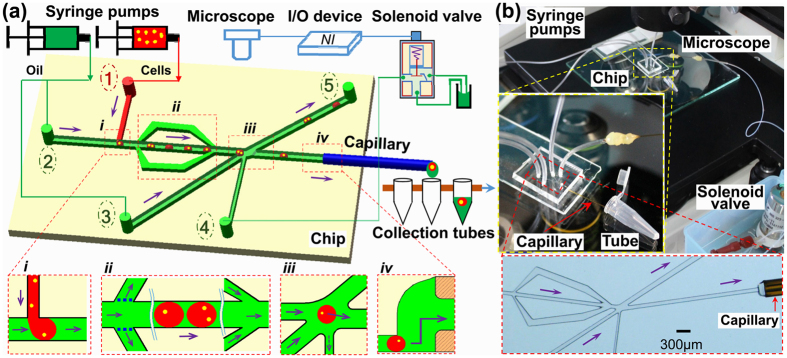
The droplet microfluidic platform for single-cell dispensing. (**a**) Schematics of the platform and single-cell isolation process including (i) cell encapsulation, (ii) droplet deceleration, (iii) sorting of single-cell droplets, and (iv) export of single-cell droplets into tubes. (**b**) Photo of the integrated microfluidic platform including the chip, syringe pumps, a microscope, a NI controlling board, a solenoid valve, and cell collecting tubes.

The droplet-based microfluidic chip consists of four functional units (Fig. 1a): (*i*) cell encapsulation in water-in-oil droplet by “T-junction” configuration, (*ii*) droplet deceleration by a branch-channel structure and inspection under microscope, (*iii*) single-cell droplet sorting by solenoid valve suction, and (*iv*) exporting of single-cell droplet into a tube via an embedded capillary interface. Cells were pumped into the chip through inlet hole 1, while oil through inlet holes 2 and 3; droplet sorting was realized by the valve through inlet hole 4; wastes were exported through outlet hole 5, and the selected single-cell droplets were exported through the capillary interface. Two types of channels were designed: the “microchannels” (~60 μm in depth) connecting inlet and outlet holes, and the “dispensing channel” (~300 μm in depth) embracing a piece of fused-silica capillary tubing as the chip-to-tube interface (chip sizes shown in Supplementary Fig. S1). Besides the chip, two syringe pumps, an inverted optical microscope, a solenoid valve device and a container (i.e. a PCR tube) were also included in the single-cell isolation workflow for cell and oil injection, droplet visual inspection, on-demand flow control and cell collection respectively (Fig. 1b).

**Figure 2:**
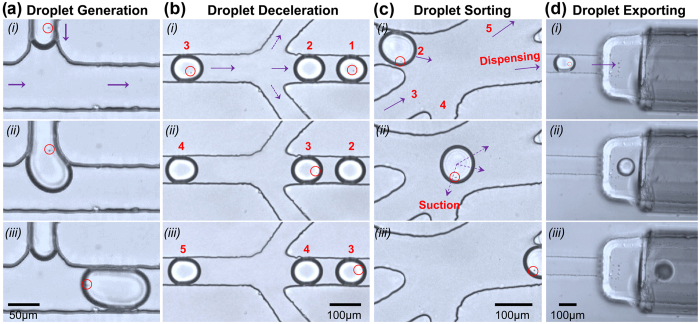
The process of single-cell droplet isolation under microscope. (**a**) Droplet generation at the “T junction” of the microchannels. (**b**) Droplet deceleration at the inspection zone. (**c**) On-demand droplet sorting by the solenoid valve. (**d**) Export of single-cell droplets through the dispensing channel. Photos were taken with time intervals 0-0.92-1.2 s, 0-0.7-1.4 s, 0-0.2-0.4 s and 0-0.24-0.44 s for (**a**), (**b**), (**c**), (**d**) respectively. Each photo showed roughly the whole microscope field of this step respectively.

The whole process was monitored in real-time under microscope step by step (Fig. 2). First we focused on the cell encapulation step, and once the droplets were produced constantly and continuously, we moved the microscope stage to observe the deceleration zone, in where cell numbers in a droplet was count. Once a single-cell droplet was identified at this step, its movement was tracked by manually moving the microscope stage until it was exported and collected. Afterwards, the microscope stage was moved back for observation of the deceleration zone in order to track another single-cell droplet, and the cycle repeated.

Specifically, cell encapsulation was the first step of the workflow. Cell suspension was injected into the chip and dispersed with a relatively slow speed (0.5 μl/min) (Fig. 2a (*i*)) into the continuous oil phase (6 μl/min) at the “T-junction” part of the channels (Fig. 2a (*ii*)). Due to the shearing effect, water-in-oil monodisperse picolitre droplets (~60 μm in diameter) were generated (Fig. 2a (*iii*)). As demonstrated previously, encapsulation of cells is random, and the distribution of cells in each droplet is dictated by Poisson statistics (*P*_*λ, k*_* = λ*^*k*^exp(*−λ*)*/k!, P*_*λ, k*_ is the probability of a droplet containing *k* number of cells, with *λ* being the mean number of cells per droplet)^15^. The maximum probability of a droplet containing one cell is 36.8% (*λ* = 1). When *λ* = 0.3, the ratio of single-cell droplet is estimated to be 22% while ratio of empty droplets is about 74%^16^,^17^. In this study, the *λ* value was set as 0.3 by adjusting the cell density to 1.5 × 10^6^/ ml to guarantee a relatively low ratio of droplets containing multiple cells. To evaluate this estimation, an independent pre-experiment was performed, droplets (diameter ≈ 60 μm) generated at the “T-junction” part was directly exported and dispensed on a hydrophilic glass slide pretreated with Pluronic^®^ F127, and were inspected under microscope. Five regions covering the whole microscopic field was randomly selected, and number of cells in each droplet inside the region was counted for estimating of the single-cell droplet ratio. The number of single-cell droplets *vs.* total droplet number was 32/148, 24/154, 44/152, 37/225 and 24/155 respectively. Overall, we showed that 19.3% of the droplets contained a single cell (Fig. S2), roughly in agreement with a Poisson distribution.

After droplet generation, the next step was to identify single-cell droplets from others under microscope (20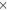 objective). Here two branch microchannels were symmetrically arranged by the two sides of the main microchannel for flow deceleration. When oil passed through branch channels, attenuation of droplets through the middle channel was achieved by the shunting effect. No droplets would enter the branch channels due to the hydrodynamic resistance of the branch channels. The rates of cell suspension and mineral oil pumped into the chip were modified to 0.5 μl/min and 6 μl/min respectively in order to obtain a good view of decelerated droplets under microscope. As shown in Fig. 2b, droplets were flowing along the channel in sequence with a distance of roughly 391.7 μm between each other (photos taken with intervals of 0.7 s). When droplets entered the inspection zone, the distance between each droplet was shortened to 133.3 μm, indicating reduction of their flow rate by 2/3. It took about 1.4 s for a droplet to pass the microscopic field (Fig. 2b), which was sufficient time for the user to observe and count the number of cells in each droplet, thus to recognize those single-cell droplets.

The third step was to separate the identified single-cell droplets from others. We previously established a capillary-tuned solenoid microvalve system which was able to induce a suction effect for on-demand microfluidic flow controlling^14^. Here, a simple method based on this effect was developed to realize single-cell droplet separation. After the screening step, all droplets were pushed to the cell sorting unit from Channel 2 (Fig. 2c (*i*)). The droplets containing multiple cells or no cell would flow into the waste Channel 5 by default, pushed by the oil flow from Channel 3 connected to the syringe pump. On the contrary, when a target single-cell droplet entered the sorting unit, the valve with response time as short as 20 ms was activated via a USB digital I/O device based on user operation, an instantaneous suction force was delivered onto the target droplet through Channel 4 connected to the valve (Fig. 2c (*ii*)), and the target droplet would flow into the dispensing channel (Fig. 2c (*iii*)). In this step, the suction force was employed as the only driving force for on-demand flow controlling, and this selection process was completed within 1 s. A buffering effect provided by the side oil had ensured that the main channel flow would not be interfered by any side-effect.

The last step was to dispense single-cell droplets one by one via a chip-to-tube interface. The interface was established by inserting a piece of fused-silica capillary tubing into the dispensing channel, while the other end acted as a simple dispensing nozzle. The capillary tubing of 20 mm in length (O.D. = 360 μm, I.D. = 150 μm) nicely fitted the dispensing channel: first, its inner diameter was large enough for the droplets (~60 μm in diameter) to pass easily; second, its short length would not induce high hydrodynamic resistance in the dispensing channel; last, its outer diameter was slightly larger than the size of the dispensing channel (~300 μm in width and ~300 μm in depth) in order to form a tight contact between the capillary and the elastic PDMS channel. Additionally, the capillary was grinded smooth at both ends and treated with the hydrophobic reagent “Aquapel” to avoid droplet fusing, splitting or trapping. After on-demand flow controlling mentioned above, a single-cell droplet was sucked to into the dispensing channel, successfully passed through the capillary interface and was collected in a tube (Fig. 2d). As the instantaneous suction force worked on the target single-cell droplet only, no other droplet would enter the dispensing channel before a second trigger. After this single-cell droplet was collected, another round of screening, identification, sorting and exporting was performed, until a second single-cell droplet was collected in a new tube. In this way, single-cell isolation with one-droplet-in-one-tube mode was achieved. Since the capillary is transparent and laterally connected with the dispensing channel, the process of dispensing can be monitored under microscope. It took only 10 min to collect 30 single-cell droplets from pumping cell suspension into the chip to exporting of the target droplets, showing the average throughput as approximately 20 s/cell (see Supplementary Video S1).

### Evaluation of the system feasibility and efficiency

**Figure 3:**
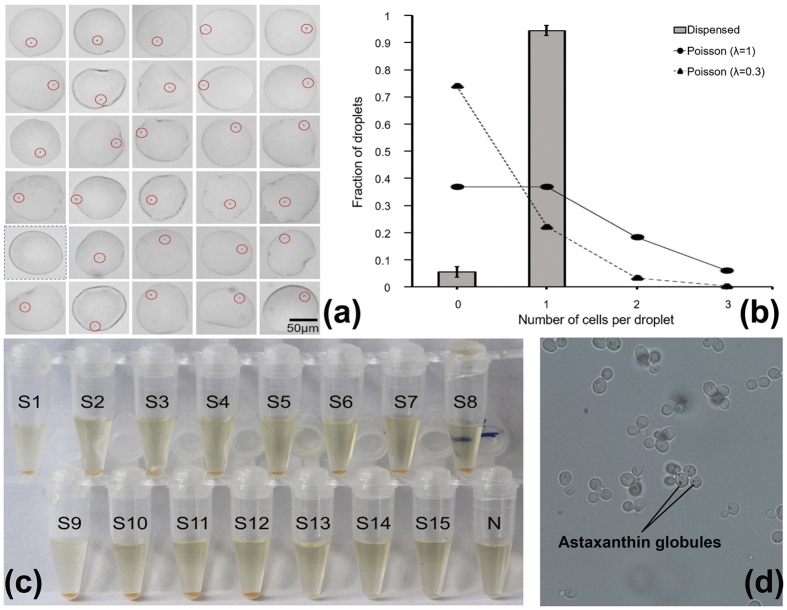
Evaluation of the single-cell droplet isolation. (**a**) Each sorted droplet was dispensed on a glass slide and the number of cells in each droplet was counted under microscope. Single cells were marked with red circles; droplets without cells were marked with blue-dotted boxes. Size and shape of droplets varied due to their expansion on the glass slide. One of the triplicates was shown. (**b**) The ratio of single-cell droplets (gray block) compared with the theoretical ratio of single-cell droplets when formed at the “T-junction” of the microchannels (*λ* = 0.3, black triangle) and the upper limit of single-cell droplets (*λ* = 1, black circle). (**c**) Result of the single-cell cultivation experiment. S: single-cell samples; N: blank droplet. (**d**) Microscopic images of *P. rhodozyma* cells from one of the tubes in (**c**).

Success rate of single-cell droplet isolation was determined by dispensing single-cell droplets on a hydrophilic glass slide pretreated with Pluronic^®^ F127 and counting the number of cells in each droplet. Dispensing of 30 single-cell droplets was performed in triplicates. The success rate of single-cell droplet isolation was 94.4 ± 2.0% (96.7%, 93.3% and 93.3% for each trial, 29/30, 28/30 and 28/30 respectively), while all other droplets were empty due to false positive selection which was probably caused by the deviation of visual inspection (Fig. 3a and Supplementary Fig. S3). By adjusting original cell concentration accroding to Poisson statistics before pumping in, the ratio of multiple-cell droplets after cell encapsulation was minimized to <4% while sufficient single-cell droplets were still available for downstream isolation. Thus the risk of false-positives induced by sorting of double/triplet-cell droplets was minimized. Notably, λ value of 0.3 was an approximate number. While droplet volume and cell density were estimated and controlled in our experiments, the values could not be measured accurately. Therefore, the ratio of single-cell droplets in different experiments could vary slightly according to the difference of droplet volume and cell density. In our experiment, λ value was set as 0.3 in order to guarantee a relatively low ratio of droplets containing multiple cells. The platform will also work if λ value is set as other numbers.

After single-cell droplet identification and isolation, this enriched ratio was significantly higher than the theoretical ratio of single-cell droplets as approximately 22% (*λ* = 0.3) at the “T-junction” of the microchannels, and even higher than the upper limit of single-cell ones as 36.8% (*λ* = 1) (Fig. 3b).

Vitality of the isolated cells was evaluated by cultivation test. Each droplet containing a single carotenoid synthesizing *Phaffia rhodozyma* ATCC 24202 cell was exported into a PCR tube. After shaking at 30 °C for 40 h, growth of *P. rhodozyma* cells was detected from 12 out of the 15 single-cell droplets (i.e. 80% success rate), as evidenced by the appearance of orange color that indicates cell growth and carotenoid synthesis, whereas no growth was seen from the blank droplet (Fig. 3c). The cells after proliferation were further checked under microscope for validation of their vitality (Fig. 3d). The failure (i.e. 20% of the droplets) might be induced by unsuccessful release of the cell from the droplet or inefficient acquisition of nutrient contents. Droplets were demonstrated to provide necessary conditions for living for different cell types including bacteria and yeast^18^. In the present study, we have also demonstrated that the single-cell isolation process introduced minimal interference to cellular vitality.

Being both facile and efficient is the main advantage of this platform. In terms of accessibility to users, only ordinary instruments such as injection pumps and a microscope, as well as low-cost commercially available devices such as a NI controlling board (~$99) and a solenoid valve (~$111) are required and the operations are quite simple.

In terms of efficiency, success rate of single-cell droplet collection of over 90% and the average throughput of 20 s/cell were demonstrated, which can be attributed to the following reasons.

First, by droplet inspection and on-demand sorting, the ratio of single-cell droplets was further enriched to >90% while false-positive sorting was avoided. Several studies have used passive methods with complicated chip design to directly increase the single-cell encapsulation efficiency to nearly 80%, including inertial microfluidic strategies making use of the inertial lift forces to focus and order cells prior to encapsulation^19^,^20^ or passive hydrodynamics approach in which droplets underwent self-sorting on the basis of purely passive hydrodynamic mechanisms^21^. Alternatively, single-cell droplets were harvested by sophisticated signal processing techniques and active microfluidic sorting methods based on dielectrophoresis^17^ or compressed pressure controlled by a valve^22^. In our study, enrichment of single-cell droplets was achieved through one-by-one screening and on-demand sorting, which is simpler and more precise. Flow deceleration and droplet identification were realized by a trifurcating branch channel structure on the chip, and on-demand sorting was realized by an easy-to-use solenoid valve suction. To the most extent, this strategy prevented multi-cell and blank droplets from entering the dispensing channel, while complicated channel fabrication and tedious operation were avoided.

In this study, with the chip designed to generate droplets with diameters of approximately 60 μm, we have demonstrated that single *Chlamydomonas* (~10 μm in diameter) or budding yeast (2~5 μm in diameter) cells can be identified and sorted reliably, therefore, this platform is believed to perform well on isolating single microbial cells of 2~10 μm in size with the speed of 20 s/cell. According to the droplet size, this platform should also be able to be applied for isolation of mammalian cells. Generally, droplet size is determined by the flow rates of the two phases in addition to the channel geometries and the viscosities of the two phases^23^. Production of droplet with smaller size would require a higher shear stress from continuous oil phase exerting on discontinuous aqueous phase, higher flow rates of both phases would thus be essential in such case. In the present study, relatively low flow rate and the branch-microchannel structure were of significant importance for single-cell droplet identification. Therefore, droplet size of ~60 μm in diameter was selected for two reasons: cells in the droplet can be observed under microscope accurately, and the flow rates (0.5 μl/min for cell suspension and 6 μl/min for mineral oil) ensured that droplets passed the deceleration part with a relative low speed so that there was sufficient time for the user to count the number of cells in each droplet.

The effect of droplet size on cell encapsulation can also be illustrated by Poisson statistics as mentioned above. For a given cell density, while droplet size increases, λ (the mean number of cells per droplet) increases accordingly. Therefore, cell density was carefully modified in our study before cell encapulation. Additional, cell vitality was not ruined by the encapulation operation nor the mineral oil as well as the Span 80 surfactant, as proved by the single-cell cultivation experiments, which was consistent with previous studies^24^,^25^.

In the future, the throughput of droplet screening as well as the identification accuracy of smaller cells (<2 μm) can be further improved by coupling digital video processing methods. For example, accuracy and speed of the droplet identification process can be increased with fluorescence labeling of targeting cells, high-resolution image recognition techniques, etc, and modification of droplet size in a broader range will also be possible.

The highly reliable droplet exporting strategy is the second key factor for the high efficiency. It was realized through a simple connection between the dispensing channel and the fused-silica capillary tubing, and was monitored in real-time under microscope. Previously, Huang *et al*.^26^ and Kasukurti *et al*.^27^ respectively used optoelectronic or optical tweezers to push human ovarian cancer cells or red blood cells out of the microfluidic device and into a collection tube; Nakamura *et al*. used a micromanipulator (CellTram Vario, Eppendorf) to pick up droplets encapsulating single environmental microbes and transfer them into PCR tubes^28^. Expensive commercial equipment played a key role for single cell export in these studies. Kim *et al*. used an air pressure driven dispensing controller to shoot out single circulating tumor cells through an exhaust needle^29^. However, the needle was arranged vertically to the fluid channel and in parallel to the objective, thus the single-cell exporting procedure could not be visualized, which might lead to imprecise collection. Comparatively, the chip-to-tube interface in our device was constructed by embedding a piece of capillary tubing into the dispensing channel, ensuring accurate monitor and control of the whole dispensing process under the microscope.

### Single-cell gene-specific analyses

Real-time qPCR or conventional PCR of a specific gene on single-cell level allows taxonomic identification of novel microorganisms or investigation of gene expression heterogeneity among populations^30^. In this prove-of-concept experiment, we demonstrated the feasibility of integrating this single-cell isolation platform with conventional protocols of gene-specific analyses using either single-cell gDNA or complementary DNA (cDNA) transcribed from single-cell RNA.

**Figure 4:**
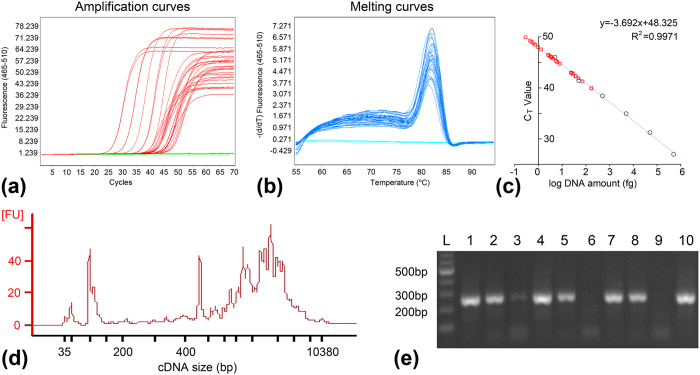
Results of the single-cell gene-specific analyses. (**a**) Amplification curves of single-cell droplet samples and standard DNA. The x axis represents PCR cycle number and the y axis represents fluorescence intensity. Only samples with C_T_ value < 60 were shown. (**b**) Melting curves of these samples. The x axis represents melting temperatures and the y axis represents the −ΔF/ΔT (change in fluorescence/change in temperature). Only samples with C_T_ value < 60 were shown. (**c**) Linear fitting of log transformed DNA concentrations *vs.* C_T_ values using standard DNA samples (dots). The single-cell droplet samples (squares) were plotted on the fitting curve by C_T_ values. (**d**) Bioanalyzer electropherograms of a representative cDNA sample, showing the size distribution of cDNA molecules. (**e**) 1.2% agarose gel electrophoresis of the partial *C. reinhardtii* 18S rRNA gene PCR products. L: DNA ladder; 1–10: ten single-cell droplet samples. Samples 6 and 9 failed to show positive amplicon in either RT-PCR or 18S rRNA gene targeted PCR.

Firstly, 60 droplets each containing a *Saccharomyces cerevisiae* cell and 20 blank droplets (as blank controls) were collected successively in each well of a 96-well plate. The cells were recovered from the droplets by centrifugation, lysed by alkaline treatment, and the released gDNA was directly used in real-time qPCR assays targeting yeast *ALG9* gene. The same qPCR assays were performed three times to evaluate the repeatability of the experiment. The second and third assays were performed on the same date while the first assay was performed separately before. Among the 60 single-cell droplets in each assay, 29, 32 and 30 showed positive amplification (C_T_ value < 60) with C_T_ value of 44.86 ± 2.86, 46.24 ± 2.91 and 46.74 ± 2.15 respectively. A C_T_ value > 60 was found in 4, 1 and 2 samples in these three assays, which were regarded as unreliable amplification. None of the 20 blank droplets in any of the three assays generated positive amplification, showing no false-positive results. The success rate was 48.3%, 53.3% and 50% (Fig. 4a and Supplementary Fig. S4a,b). No non-specific amplification was observed in any assay as indicated by the melting curves (Fig. 4b and Supplementary Fig. S4c,d). Furthermore, as a positive control for the DNA amplification, a standard curve was generated using six DNA standards ranging from 5 to 5×10^4^ fg (the DNA standard of 0.5 fg failed to show valid amplification). The correlation coefficient (R^2^) was 0.9971, 0.9875 and 0.9901, suggesting the high amplification efficiency of the PCR reactions (Fig. 4c and Supplementary Fig. S4e,f)^31^ and successful amplification of the standard DNA equivalent to the DNA in a single yeast cell (~10 fg) was demonstrated. Therefore, both the success rate of PCR amplification from single cells, risk control of contamination and the standard deviation of the C_T_ value among the various single-cell reactions were comparable to previous reports^13^,^32^,^33^. For the three repeating qPCR assays, we saw variation of absolute values between different plates, which is frequently reported in studies with real-time qPCR, even though the measurements were technically carried out with identical procedures^34^. Therefore, comparison of different samples were usually carried out within a plate. In our study, we have showed that the standard deviation within the plate in all cases was consistent, indicating the reliability of the isolation method in coupling with common real-time qPCR analysis. Overall, the reaction failures might be attributed to inefficient cell lysis, inaccessibility of genomic DNA, or suboptimal PCR performance. Therefore, the amplification efficiency can be improved in future studies by more sufficient cell lysis, selection of primers and optimization of PCR conditions, etc.

The advantage of our method in ease of operation is also apparent. In previous studies, Shi *et al*.^33^ and Leung *et al*.^13^ had respectively performed single-cell qPCR reactions in adhering droplet arrays or on-chip chambers, and showed the advantages of the microfluidic devices in cost, throughput, and precision compared with other approaches as micromanipulators^35^, FACS^36^, etc. The shortcoming of their studies lay in the need for expensive integrated equipment or sophisticated chip architecture, i.e. a pick-and-place single-cell manipulation robot, or a programmable microvalve based microfluidic chip. In this study, such tedious operations were avoided by collecting single-cell droplets in PCR tubes through a capillary interface, and the real-time qPCR reactions were performed with an ordinary fluorescent thermal cycler.

Secondly, 10 droplets each containing a single *Chlamydomonas reinhardtii* cell and two blank droplets (as blank controls) were prepared for RNA analysis. Each single cell was collected as above and lysed with protection of RNase inhibitor. Reverse transcription PCR (RT-PCR) was performed to generate cDNA from the released single-cell RNA. Double-stranded cDNA was successfully generated from 8 out of the 10 single-cell droplets (accounting for 80%) with cDNA yield of 435.1 ± 136.3 ng. No cDNA was detected in the other two samples or the two blank controls by the Qubit dsDNA HS Assay (detection limitation of 10 pg/μl). The generated cDNA profile had a main peak at ~1–2.5 kb, with a small number of fragments of primer dimers (Fig. 4d). Both the cDNA yield and size distribution were in accordance with cDNA generated from bulk cell culture with conventional methods, indicating that integrity of RNA molecules was well preserved during cell isolation, which is important for downstream RT-PCR and gene expression profiling^37^.

Furthermore, PCR targeting the 18S rRNA gene of *C. reinhardtii* was performed using the cDNA as template for verification. Positive amplicons were obtained from all eight positive cDNA samples (Fig. 4e). Following PCR, the amplicons from each of the 8 positive reactions were eluted for further amplification and Sanger sequencing. All single *C. reinhardtii* cells were correctly identified by the sequence of the 18S rRNA gene. These results showed acceptable yield of cDNA followed by efficient amplification of a marker gene from a single cell, thus the present single-cell isolation method can be used for analysis of gene expression heterogeneity among microbial populations.

### Single-cell whole genome sequencing

**Figure 5:**
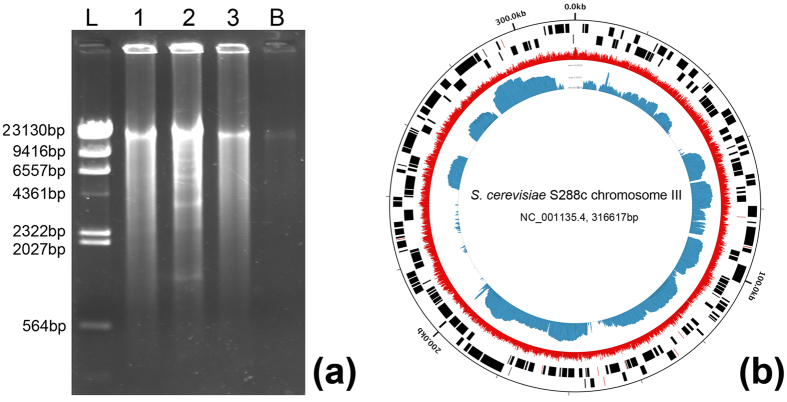
Results of single-cell genomic DNA amplification and sequencing. (**a**) 0.8% agarose gel electrophoresis of MDA products. L: DNA ladder; 1–3: three representative single-cell droplet samples; B: blank droplets. (**b**) The Circleator figure of reads aligned to the *S. cerevisiae* S288c genome. From outside to inside: coordinate labels of the S288c genome; forward and reverse strand genes of the S288c genome; percent GC content of the sequencing reads of the single-cell sample (shown in red); read coverage of the single-cell sample (shown in blue). The chromosome name, NCBI accession and size of the chromosome were specified in the center. Only the Chromosome III of the yeast was shown here, as an example (additional details in the Supplementary Information).

Single-cell genome sequencing can provide a comprehensive functional landscape of individual cells. Here we amplified the gDNA from ten single *S. cerevisiae* cells respectively using the Multiple Displacement Amplification (MDA) method. Positive amplicons were observed from all ten single-cell droplets. Size of the amplified gDNA was ~10–20 kb (Fig. 5a). The gDNA yield from a single cell was 820.6 ± 293.5 ng, ranging from 532.4 ng to 1461.6 ng, which was sufficient for subsequent experiments such as multiple PCRs or sequencing library preparation. Oppositely, only trace amounts of amplicon were seen from the blank droplets (DNA yield < 10 ng), which might be caused by biased amplification of primer dimers. PCRs targeting the 26S rRNA gene of *S. cerevisiae* and the bacterial 16S rRNA gene were performed on all samples, positive amplification of the *S. cerevisiae* gene fragment was achieved in all single-cell samples and no blank samples, while no bacterial contaminant was seen in any of the samples.

Next, amplified gDNA from one single cell was used for library preparation and sequencing on Illumina HiSeq 2500. Totally, 3714.46 Mb of high quality sequencing data, in the form of 12,381,541 × 2 paired-ended reads, was obtained with a read length of 150 bp at both ends and an average insert size of 300 bp. Among them, 99.2% of the read pairs were successfully aligned to the reference genome of *S. cerevisiae* S288C with theoretically 305-fold coverage of the genome. These reads distributed unevenly across the whole nuclear genome (organized in 16 chromosomes) as well as the mitochondrial DNA (Fig. 5b and Supplementary Fig. S5). The genomic assembly was then performed using IDBA_UD^38^, producing 2,017 contigs with total length of 5.2 Mbp, which represented 43.3% of the whole yeast genome. These results are comparable with past studies which reported that single-cell genome sequences recovered from environmental microorganisms are 40–55% complete on average, ranging from a few percent to greater than 90%^39^. The non-uniform and relatively low coverage may be attributed to incomplete cell lysis, partial degradation of the template DNA, or bias of the MDA reaction, and could be improved by combined assemblies of closely related single cells in future studies. Moreover, our results demonstrated that existence of mineral oil would not affect downstream DNA/RNA amplification and analysis, which was also supported by a similar study^25^.

Therefore our single-cell isolation platform is capable of preparing single microbial cells for producing high yields of cDNA and gDNA that are comparable in quality with those from bulk culture and ready for mRNA-Seq and whole genome sequencing.

## Conclusions

This work presented a facile and efficient droplet microfluidic platform for single microbial cell isolation without sophisticated facilities. By integrating single-cell droplet encapsulating, sorting and dispensing, single microbes could be dispensed with >90% success rate in the mode of one-cell-in-one-tube. The dispensed single cells can be readily coupled to single-cell cultivation, gene-specific analysis and genome sequencing. There is also possibility to improve the throughput of this strategy with digital video processing methods. This platform should encourage and accelerate wider application of single-cell analysis technologies in microbiology labs.

## Methods

### Reagents and materials

All reagents were purchased from Sigma Aldrich (USA) except specified otherwise. Budding yeast strains *S. cerevisiae* BY4742 and *P. rhodozyma* ATCC 24202 were maintained and inoculated with Yeast Extract-Peptone-Dextrose (YPD) media^40^. The microalga *C. reinhardtii* strain CC124 was cultured with Tris-Acetate-Phosphate (TAP) media as described previously^41^. The *P. rhodozyma* cells show orange color in tubes and under microscope as they synthesize carotenoid astaxanthin during growth^42^. Details of cultivation procedures were provided in the Supplementary Information. Cell cultures at stationary phase were harvested and processed right before isolation experiments.

### Chip fabrication

The microfluidic chip consisted of a main poly (dimethylsiloxane) (PDMS) layer (15 mm×20 mm×3 mm) with embedded channels, which was sealed on a PDMS-coated glass substrate (75 mm×25 mm ×1 mm). Chip structure was designed with AutoCAD 2007 (Autodesk, USA) and printed on two photolithography masks: Mask-A with pattern of all microchannels and inlet/outlet holes; Mask-B with that of the dispensing channel (Supplementary Fig. S1). Fabrication of master mold was achieved by a two-step soft-lithographic technique.

*Firstly*, a flat layer of 60 μm in thickness was created by spinning 2 ml of SU-8 3025 photoresist (MicroChem, USA) on a Ф7.5 cm silicon wafer at 500 rpm for 10 s followed by 1250 rpm for 30 s and soft baking (65 °C for 10 min, 95 °C for 20 min and cooling down). After covering Mask-A on the layer, the microchannels were fabricated by exposure under UV (365 nm, 9 mJ/cm^2^ for 25 sec), post baking (same as above), and final development (rinsing the mold in SU-8 developer whiling shaking for 3 min). The wafer was then washed with isopropanol and dried with nitrogen (Supplementary Fig. S6a).

*Secondly*, a second layer of 300 μm in thickness was created with SU-8 2075 photoresist (MicroChem, USA). As 300 μm is beyond the photoresist thickness limitation, the operations of spinning 2 ml of SU-8 2075 at 500 rpm for 10 s followed by 1625 rpm for 30 s and soft baking were repeated twice. The dispensing channel was fabricated in the same way as above after aligning Mask-B on this layer (Supplementary Fig. S6b). After the mold fabrication, PDMS mixed with catalyst (Dow Corning, USA) at a ratio of 10:1 (w/w) was degassed to remove bubbles, poured onto the master mold, degased again, cured at 80 °C for 2 h, and peeled away (Supplementary Fig. S6c). Inlet and outlet holes were punched into the layer at different ends of channels using a needle with a tip diameter of 10 mm.

*Thirdly*, a PDMS-coated glass substrate was prepared by pouring 1-mm-thick PDMS layer onto a clean flat glass slide. The PDMS layer and the glass substrate were then exposed to oxygen plasma (PLASMA-PREEN II-862; Plasmatic systems, Inc., USA) for 30 s and bonded to form a permanent seal by baking at 80 °C for 24 h (Supplementary Fig. S6d). A chip-to-tube capillary interface was assembled by inserting one end of a piece of fused silica capillary tubing (20 mm in length, outer diameter (O.D.) = 360 μm, inner diameter (I.D.) = 150 μm; Labsmith, USA) into the dispensing channel of the PDMS chip. A water-resistant agent “Aquapel” (PPG Co., USA) is grafted to the capillary tube to achieve inner surface hydrophobicity before assembly.

### Microfluidic control

The chip was fixed on an inverted microscope (IX71; Olympus, Japan) with 20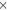 objective. Oil phase and aqueous phase were continuously injected into the microchannels via separate inlet holes by two syringe pumps (LSP01-2A, Longer Pumps, China) through PEEK tubings (O.D. = 0.0625 inch, I.D. = 0.04 inch; Upchurch Scientific, USA). A solenoid valve (Three-Way Direct Lift Solenoid Valve; Cole-Parmer, USA) module was set up and controlled as described in our previous study (Supplementary Fig. S7)^14^. Specifically, the “Comm.” port of the valve was connected to the corresponding hole of the chip through PEEK tubing with a piece of fused-silica capillary tubing (30 mm in length, O.D. = 360 μm, I.D. = 150 μm), while the “N.C.” and “N.O.” ports were connected to oil pool through PEEK tubings. The valve with response time as short as 20 ms was activated via a USB digital I/O device (USB-6501; National Instruments, USA) linked to a computer with a homemade script using LabVIEW (National Instruments, USA) based on user operation, which would then deliver an instantaneous suction force onto the target droplet. Within an actuation cycle of the valve (OFF → ON → OFF), a small volume of liquid would be sucked into the “Comm.” port, indicating a “suction” effect on the valve connected microchannel. The controllability of the suction process was evaluated by both computational fluid dynamics simulation and on-chip experiments. In this previous study, we have demonstrated that the suction volume could be easily manipulated by adjusting the energized valve duration between 25 and 100 ms, and the valve actuation of 60 ms was suitable for a target cell to be sucked and collected^14^. In the present study, the valve duration of 60 ms was applied accordingly.

### Single-cell isolation experiments

Before the experiments, surfaces of all equipment (objective platform, pump, solenoid valve, etc.) were cleaned with DNA AWAY^TM^ (Molecular BioProducts, USA). All consumables, including DNase/RNase-free ddH_2_O (Millipore-Q-plus water purification system; Millipore, USA), oil, the chip and capillaries, syringes, PEEK tubings, and droplet collecting tubes, were treated with Stratalinker 2400 UV Crosslinker (Stratagene, USA) at 254 nm for 30 min to inactivate any contaminant DNA before use^43^.

Cell cultures harvested at stationary phase were diluted to a final cell density of 1.5 × 10^6^/ml before use to encapsulate theoretically λ = 0.3 cells in droplets with diameter of 60 μm. As estimated by the Poisson statistics (*P*_*λ, k*_* = λ*^*k*^exp(*−λ*)*/k!, P*_*λ, k*_ is the probability of a droplet containing *k* number of cells, with *λ* being the mean number of cells per droplet)^15^, this choice of cell density would result in 74.08% of the droplets containing no cells, 22.22% containing a single cell, 3.3% containing two cells and 0.38% containing more than two cells at the stage of droplet occupancy^17^. Specifically, in single-cell isolation experiments for nucleic acid analysis, cells were washed three times and resuspended in ddH_2_O with the same cell density of 1.5 × 10^6^/ml before use.

To provide continuous supply of cells, 1 ml of cell suspension and 1 ml of mineral oil containing 2.5% (w/w) Span80 surfactant were individually sucked with a syringe and pumped into the chip via different holes with speed of 0.5 μl/min and 6 μl/min respectively (Fig. 1a). Cell-encapsulating droplets (~60 μm in diameter) were generated continuously and tracked under microscope while they moved in the channel. Once a single-cell droplet was observed, the solenoid valve was actuated via manual operation. As a result, the droplet was sucked into the dispensing channel, flew along the capillary tubing, and was dispensed neatly onto a hydrophilic glass slide pretreated with Pluronic^®^ F127 (which is to ensure that the dispensed water-in-oil droplets spread out on the glass slide and high-quality pictures of single-cell droplets can be captured), or was collected at the end of the capillary into a low-binding 0.2-ml PCR tube (MAXYMum Recovery™; Axygen, USA) or a well of a 96-well PCR plate for subsequent single-cell sequencing or single-cell cultivation experiments.

During the whole procedure from pumping to single-cell droplet isolation, cell suspension and oil were pumped into the chip with constant speed to ensure continuous cell encapsulation. In our experiments, 1 ml was enough to maintain continuous supply of cells with constant flow rate for more than 10 min, which was sufficient time for isolation of 30 single-cell droplets. While the cell density would decrease slightly due to adhering to the channel wall, sufficient cell supply was ensured.

### Single cell cultivation

Fifteen droplets each harboring a single *P. rhodozyma* cell and one droplet with no cell were collected individually in a 0.2-ml PCR tube containing 100 μl YPD broth as above. Cells were recovered from the droplets by votex and centrifugation at approximately 4,000 rpm as described in literature^44^. All tubes were cultivated at 30 °C aerobically while shaking at 180 rpm.

### Real-time quantitative PCR (qPCR) of single-cell DNA

Eighty wells of a 96-well PCR plate were each filled with 1 μl of PBS buffer (pH 8.0) before single-cell droplet collection. Sixty droplets each with a single *S. cerevisiae* cell and twenty blank droplets were then collected successively in these wells. Cells were recovered from the droplets as described above and were lysed by adding 1.5 μl of buffer D2 (REPLI-g Single Cell Kit; Qiagen, USA) containing 0.08 mol/L dithiothreitol (DTT) and incubation at 65 ^o^C for 10 minutes, followed by neutralization with 1.5 μl of Stop Solution (REPLI-g Single Cell Kit; Qiagen, USA). The volume of droplet after cell lysis in each of the wells was about 5 μl. A 162-bp fragment of yeast *ALG9* gene was then amplified using the cell lysates as template (Supplementary Table S1)^45^. The remaining sixteen wells on the same plate were used for standard curve measurement using DNA extracted from yeast bulk culture. Each 20-μl reaction was performed and monitored on a LightCycler 480 Real-Time PCR System (Roche Applied Science, USA). Experimental details were provided in the Supplementary Information.

### Reverse transcription PCR (RT-PCR) of single-cell RNA

Ten droplets each harboring a single *C. reinhardtii* cell and two droplets with no cells were collected individually in a 0.2-ml PCR tube containing 2 μl of freshly prepared lysis buffer^34^. Cell lysis, reverse transcription and PCR purification were performed successively for double-stranded cDNA synthesis. The generated cDNA samples were electrophoretically analyzed with an Agilent 2100 Bioanalyzer (Agilent Technologies, USA) and quantified with the Qubit 2.0 instrument (Life Technologies, USA). Partial 18S rRNA gene of *C. reinhardtii* was amplified using the cDNA as template on an Eppendorf thermal cycler (Eppendorf AG, Germany) (Supplementary Table S1). Experimental details were shown in the Supplementary Information.

### Single-cell whole-genome amplification and sequencing

Ten droplets each harboring a single *S. cerevisiae* cell and two empty droplets were collected individually in a 0.2-ml PCR tube containing 1 μl of PBS buffer. Cells were lysed and neutralized as described above. Total gDNA was amplified from all single-cell droplets with the Multiple Displacement Amplification (MDA) approach with RepliPHI™ Phi29 DNA Polymerase (Epicentre, USA) according to the manufacturer’s protocol. The same procedure was performed on all blank droplets as control. Genomic DNA amplicons were examined for integrity by 0.8% agarose gel electrophoresis. PCR assays targeting 26S rRNA gene of *S. cerevisiae* and bacterial 16S rRNA gene were performed to verify the positive amplification of yeast gDNA and no amplicon from bacterial DNA contaminants^46^,^47^. Amplified gDNA from one single-cell droplet was sequenced on an Illumina HiSeq2500 platform with 2 × 150PE format. All generated reads were aligned to the reference genome of *S. cerevisiae* S288C (GenBank: GCF_000146045.2) using the software Bowtie 1.1.2^48^. In parallel, the reads were quality-controlled and assembled using IDBA_UD 1.1^38^. Sequence data was deposited in the NCBI Sequence Read Archive (SRP067104). Experimental details were provided in the Supplementary Information.

## Additional Information

**How to cite this article**: Zhang, Q. *et al*. Development of a facile droplet-based single-cell isolation platform for cultivation and genomic analysis in microorganisms. *Sci. Rep.* **7**, 41192; doi: 10.1038/srep41192 (2017).

**Publisher's note:** Springer Nature remains neutral with regard to jurisdictional claims in published maps and institutional affiliations.

## Supplementary Material

Supplementary Information

Supplementary Video S1
