# Supplementary material for: Development of a facile droplet-based single-cell isolation platform for cultivation and genomic analysis in microorganisms
Source: Sci Rep. 2017 Jan 23;7:41192. doi: 10.1038/srep41192 (PMC5253770; doi:10.1038/srep41192)
Supplement: Supplementary Information [file srep41192-s1.pdf]

Supplementary Information for

Development of a facile droplet-based single-cell isolation platform for cultivation and genomic analysis in microorganisms

Qiang Zhang[1,+], Tingting Wang[1,+], Qian Zhou[1,2], Peng Zhang[1], Yanhai Gong[1], Honglei Gou[1], Jian Xu[1,*] and Bo Ma[1,*]

[1] Single-Cell Center, CAS Key Laboratory of Biofuels and Shandong Key Laboratory of Energy Genetics, Qingdao Institute of Bioenergy and Bioprocess Technology, Chinese Academy of Sciences, Qingdao, 266101, China

[2] Key Laboratory for Sustainable Development of Marine Fisheries, Ministry of Agriculture, Yellow Sea Fisheries Research Institute, Chinese Academy of Fishery Sciences, Qingdao, 266071, China

[+] These authors contributed equally to this work.

*Correspondence: mabo@qibebt.ac.cn; xujian@qibebt.ac.cn

Supplementary Materials and Methods

Materials

Budding yeast strains *Saccharomyces cerevisiae* BY4742 and *Phaffia rhodozyma* ATCC 24202 were maintained on YPD agar plate (10 g/l yeast extract, 20 g/l peptone, 20 g/l D-glucose and 15 g/l agar) at 4 ℃. Before each cell isolation trial, a single colony was selected and inoculated in YPD broth (10 g/l yeast extract, 20 g/l peptone and 20 g/l D-glucose) and grew at 30 ℃ while shaking at 180 rpm overnight. The *P. rhodozyma* cells show orange color in tubes and under microscope as they synthesize carotenoid astaxanthin during growth[1]. The microalgae strain *Chlamydomonas reinhardtii* CC124 (http://chlamycollection.org/) was maintained on TAP (Tris-acetate-phosphate) plate and inoculated in TAP broth under continuous lighting (approximate 150 μmol photons/m^2/s) at 25 ℃ and was bubbled with air to ensure mixing and prevent settling[2]. Cells were harvested at stationary phase.

Real-time qPCR of single-cell DNA

Eighty well of a 96-well PCR plate were each filled with 1 μl of PBS buffer (pH 8.0) before single-cell droplet collection. Sixty droplets each with a single *S. cerevisiae* cell and twenty blank droplets were then collected successively in these wells. Cells were lysed by adding 1.5 μl of buffer D2 (REPLI-g Single Cell Kit; Qiagen, USA) containing 0.08 mol/L dithiothreitol (DTT) and was incubated at 65 ^{o}C for 10 min, followed by neutralization with 1.5 μl of Stop Solution (REPLI-g Single Cell Kit; Qiagen, USA). The volume of droplet after cell lysis was about 5 μl.

Genomic DNA (gDNA) was extracted from *S. cerevisiae* culture at stationary stage with E.Z.N.A.® Yeast DNA Kit (Omega Bio-Tek, USA) and diluted to a series of seven final concentrations as 0.5, 5, 50, 500, 5000, 5×10^4 and 5×10^5 fg/μl for the establishment of a standard curve. A 162-bp fragment of yeast *ALG9* gene was amplified using primer pair ALG9-F and ALG9-R (Supplementary Table S1 online)[3]. Each 12-μl reaction contained 6 μl of 2× Reaction Mix (LightCycler FastStart DNA Master SYBR Green I; Roche, USA), 25 pmol of each primer and 1 μg of BSA (Invitrogen, USA). The 5-μl lysate or 1 μl of DNA standard samples of concentration gradient was used as template. Reactions for standards were performed in duplicate. The left two wells were used as negative controls and 1 μl of DNase/RNase-free water was used as template in each reaction.

The qPCR reactions were performed on a LightCycler 480 Real-Time PCR System

(Roche Applied Science, USA). The thermocycling program consisted of heating at 95 ℃ for 10 min, followed by 70 cycles of 15 s at 95 ℃ and 1 min at 55 ℃. After all cycles, amplification specificity was monitored via melting curve analysis of PCR end products by increasing the temperature at a rate of 0.5 °C per 5 s from 60 to 95 °C with continuous fluorescence collection.

Reverse transcription PCR (RT-PCR) of single-cell RNA

RT-PCR was performed as described in previous literature[4]. Lysis buffer was prepared by adding 1 μl of RNase inhibitor (40 U/μl, Clontech, USA) to 19 μl of a 0.2% (vol/vol) Triton X-100 solution right before experiment and shortly stored at 4 ℃. Ten droplets each with a single *C. reinhardtii* cell and two blank droplets were collected individually in a 0.2-ml PCR tube containing 2 μl of lysis buffer. After adding 1 μl of Oligo-dT_{30}VN primer (10 μM, 5'–AAGCAGTGGTATCAACGCAGAGTACT30VN) and 1 μl of dNTP mix (10 mM each; Fermentas, Lithuania) to each tube, cell lysis was accomplished by incubation at 72 ℃ for 3 min. First-strand and second-strand reverse transcription reactions were performed successively for double-stranded cDNA synthesis on an Eppendorf thermal cycler (Eppendorf AG, Germany) as described. PCR cycle numbers for first-strand and second-strand cDNA synthesis were set as 10 and 25 respectively as suggested in the literature. The generated cDNA samples were purified with Ampure XP beads with 1:1 ratio, followed by electrophoretically analysis with an Agilent 2100 Bioanalyzer (Agilent Technologies, USA). DNA concentration was further estimated with the Qubit 2.0 instrument applying the Qubit dsDNA HS Assay (Life Technologies, USA). A primer pair named Cr-18S-F and Cr-18S-R (Supplementary Table S1 online), specifically targeting a 257-bp 18S rRNA gene fragment of *C. reinhardtii* CC124, was designed based on its 18S rRNA gene sequence (Genbank EU925397)[5] using Primer 5.0 software (Primer-E Ltd., UK) for the verification of positive amplification of *C. reinhardtii* cDNA. Each 25-μl reaction contained 2.5 μl of 10× Reaction Buffer, 1U of Taq (Takara, China), 1 μl of dNTP mix (10 mM each; Fermentas, Lithuania), 25 pmol of each primer and 1 μl of cDNA product. The PCR reactions were performed on an Eppendorf thermal cycler (Eppendorf AG, Germany) The amplicon was visualized with electrophoresis on a 1.2% agarose gel, purified and sequenced with the ABI 3700™ automated DNA sequencer (Applied Biosystems, USA).

Single-cell whole genome amplification (WGA) and sequencing

Ten droplets each with a single *S. cerevisiae* cell and two blank droplets were collected individually in a 0.2-ml PCR tube containing 1 μl of PBS buffer. Cells were lysed and neutralized as described above. After neutralization, all droplet samples were amplified with RepliPHI™ Phi29 DNA Polymerase (Epicentre, USA). The 15 μl reaction contained 1.5 μl of 10× reaction buffer, 50 μM random hexamers with phosphorothioate modification of the two 3’-terminal nucleotides (IDT DNA, USA)[6], 0.4 mM dNTP, 5% DMSO (Sigma, USA), 10 mM DTT (Sigma, USA) and 100 U Phi29[7]. A master mix of MDA reagents as above was assembled in a Safe-Lock 1.5 ml Eppendorf tube and UV treated on ice in the Stratalinker 2400 UV Crosslinker (Stratagene, USA) at 254 nm for 30 min in order to eliminate possible amplification of contaminating DNA as described before[8]. Afterwards, MDA reactions were run on an Eppendorf thermal cycler (Eppendorf AG, Germany) at 30 ℃ for 16 h followed by termination at 65 ℃ for 10 min. MDA products were checked by 0.8% agarose gel electrophoresis, followed by dilution of 1000 fold in DNase/RNase-free water and 1 μl was used as template DNA in PCRs for validation. Primer pair NL1 /NL4 targeting partial 26S rRNA gene of *S. cerevisiae*[9] and 927F/1492R targeting partial bacterial 16S rRNA gene[10] (Supplementary Table S1 online) were used to verify the positive amplification of yeast gDNA and no amplicon from bacterial DNA contaminant. PCR reactions were performed and products were checked as above.

For sequencing analysis, the single-cell DNA amplicon was digested with S1 nuclease (Takara Bio Inc., Japan) in order to remove single-stranded DNA[11] according to the manufacturer’s instructions followed by phenol-chloroform purification. Sequencing library was prepared with Accel-NGS 2S Plus DNA library Kit (Swift Biosciences, USA) with 50 ng amplified gDNA as input, and sequenced on Illumina HiSeq2500 platform with 2×150PE format. All generated reads were aligned to the reference genome of *S. cerevisiae* S288C (GenBank: GCF_000146045.2) using the software Bowtie 1.1.2[12], and assembled using IDBA_UD 1.1[13]. The percent GC-content of reads aligned to each chromosome and the read coverage were calculated in nonoverlapping windows of length 100 bp respectively. Read coverage was calculated from read mapping information and log-transformed before use. The genome coverage was then visualized by a Perl application Circleator[14].

Supplementary Table and Figures

Supplementary Table S1 Primer pairs used in this study

Targeting gene	Primer name	Sequence(5’-3’)	Length of amplicon (bp)	Reference
ALG9 gene of *S. cerevisiae*	ALG9-F	CACGGATAGTGGCTTTGGTGAACAATTAC	162	3
	ALG9-R	TATGATTATCTGGCAGCAGGAAAGAACTTGGG		
partial 18S rRNA gene of *C. reinhardtii* CC124	Cr-18S-F	GGGCATTCGTATTCCGTTGT	257	-
	Cr-18S-R	TTTCAGCCTTGCGACCAT		
partial 26S rRNA gene of *S. cerevisiae*	NL1	GCCATATCAATAAGCGGAGGAAAAG	520	9
	NL4	GGTCCGTGTTTCAAGACGG		
partial bacterial 16S rRNA gene	927F	AAACTYAAAKGAATTGRCGG	565	10
	1492R	GGTTACCTTGTTACGACTT		

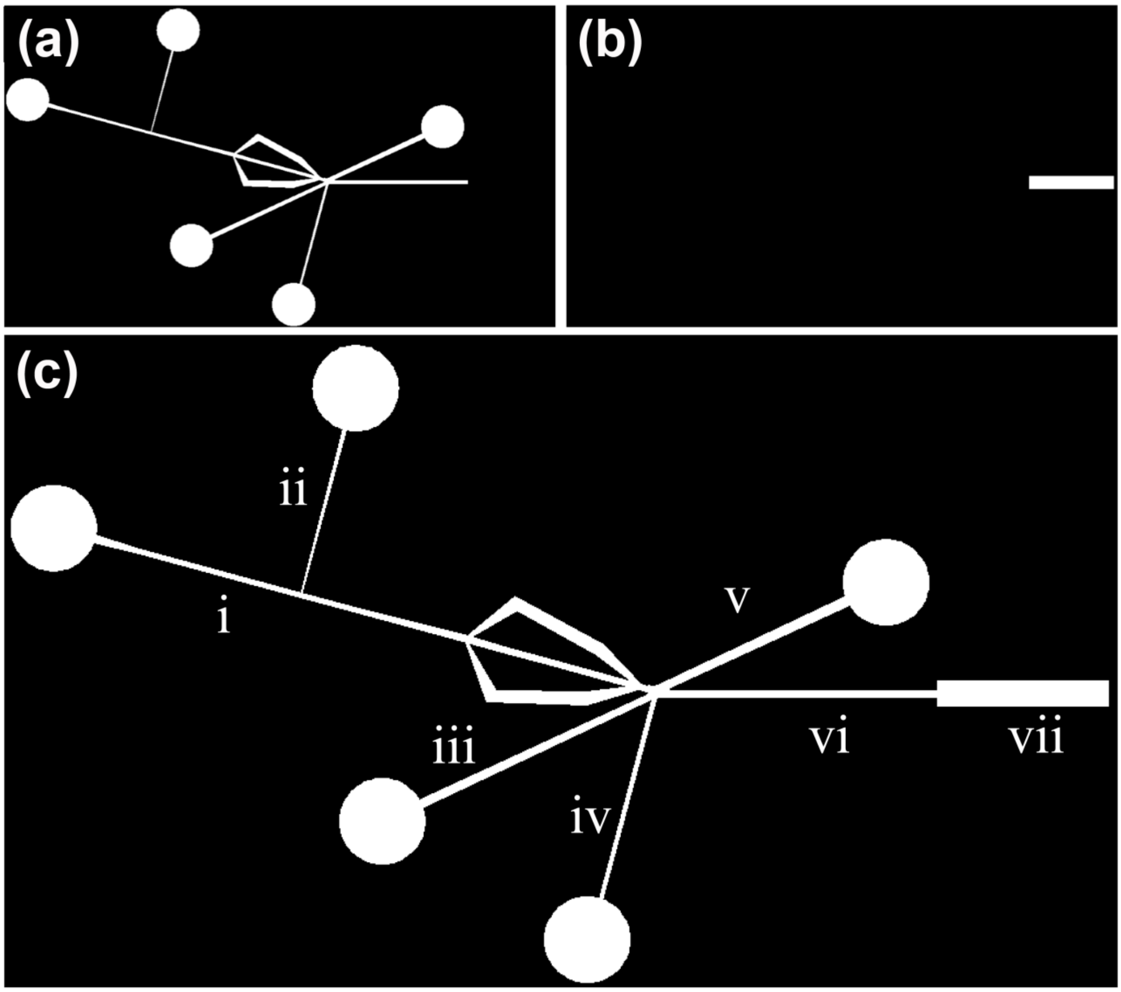

Supplementary Fig. S1 Photolithography masks showing structures of all channels and inlet/outlet holes with a resolution of 12000 dpi. (a) Mask A with pattern of all microchannels and inlet/outlet holes for aims of importing oil or aqueous phase, connecting the solenoid valve and exporting wastes. (b) Mask B with pattern of the dispensing channel. (c) Alignment of both masks. Design width of the channels are as below: i: 70 μm; ii: 35 μm; iii: 90 μm; iv: 40 μm; v: 110 μm; vi: 100 μm; vii: 300 μm.

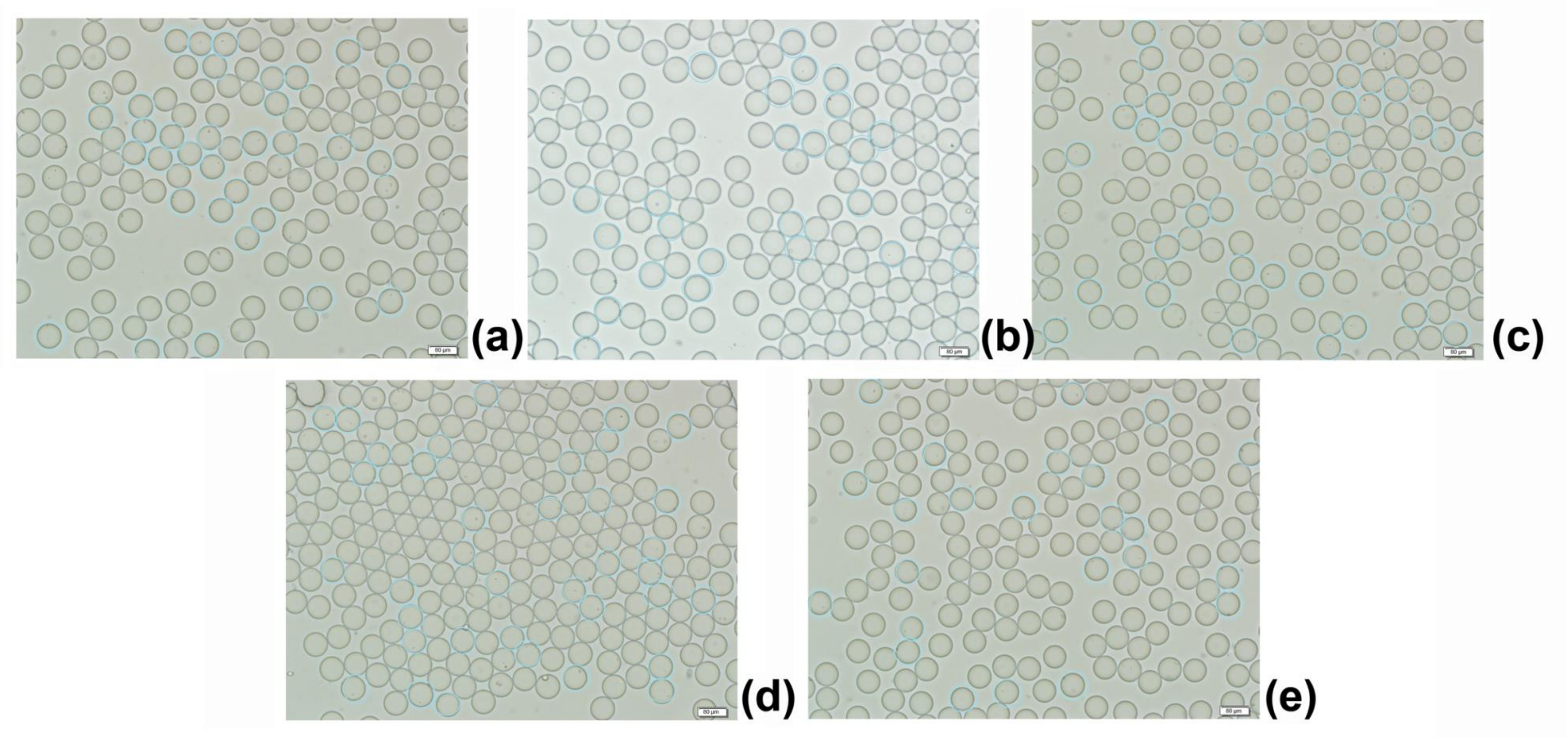

Supplementary Fig. S2 Evaluation of the Poisson statistics. Droplets (diameter ≈ 60 μm) generated at the "T-junction" section was directly exported, dispensed on a hydrophilic glass slide pretreated with Pluronic® F127 and inspected under microscope. Five regions of the whole microscopic field was randomly selected, and number of cells in each droplet of the region was counted for estimating of the single-cell droplet ration. The number of single-cell droplets *vs.* total droplet number was 32/148 (a), 24/154 (b), 44/152 (c), 37/225 (d) and 24/155 (e) respectively. Therefore overall ratio of single-cell droplets was 19.3%.

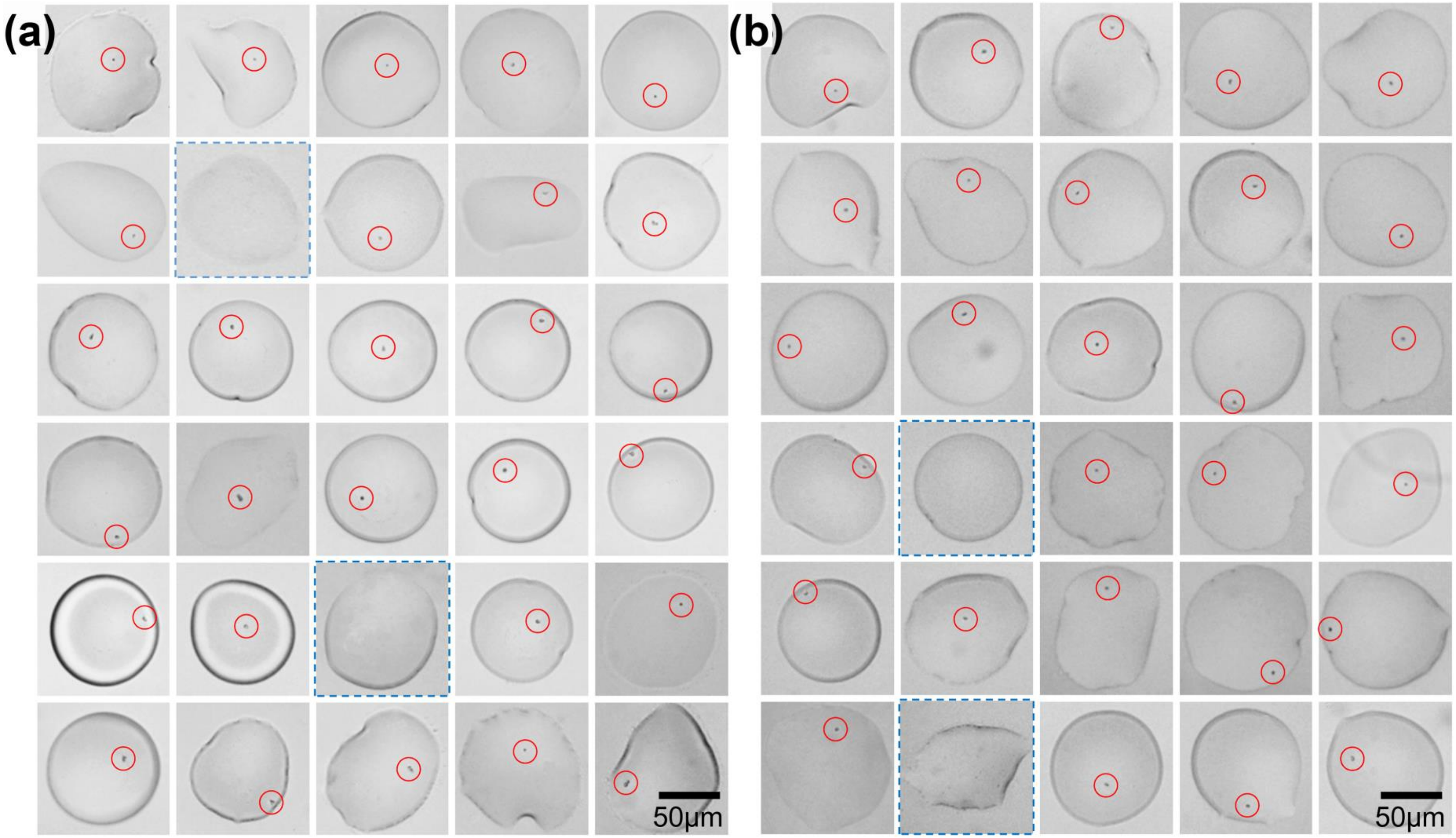

Supplementary Fig. S3 Validation of the single-cell droplet isolation. All sorted droplets were dispensed on a glass slide; cell number in each droplet was inspected under microscope. Single cells were marked with red circles; droplets without cells were marked with blue dotted boxes. Shape of droplets varied due to different expansion on the glass slide. Two of the triplicates were shown in (a) and (b).

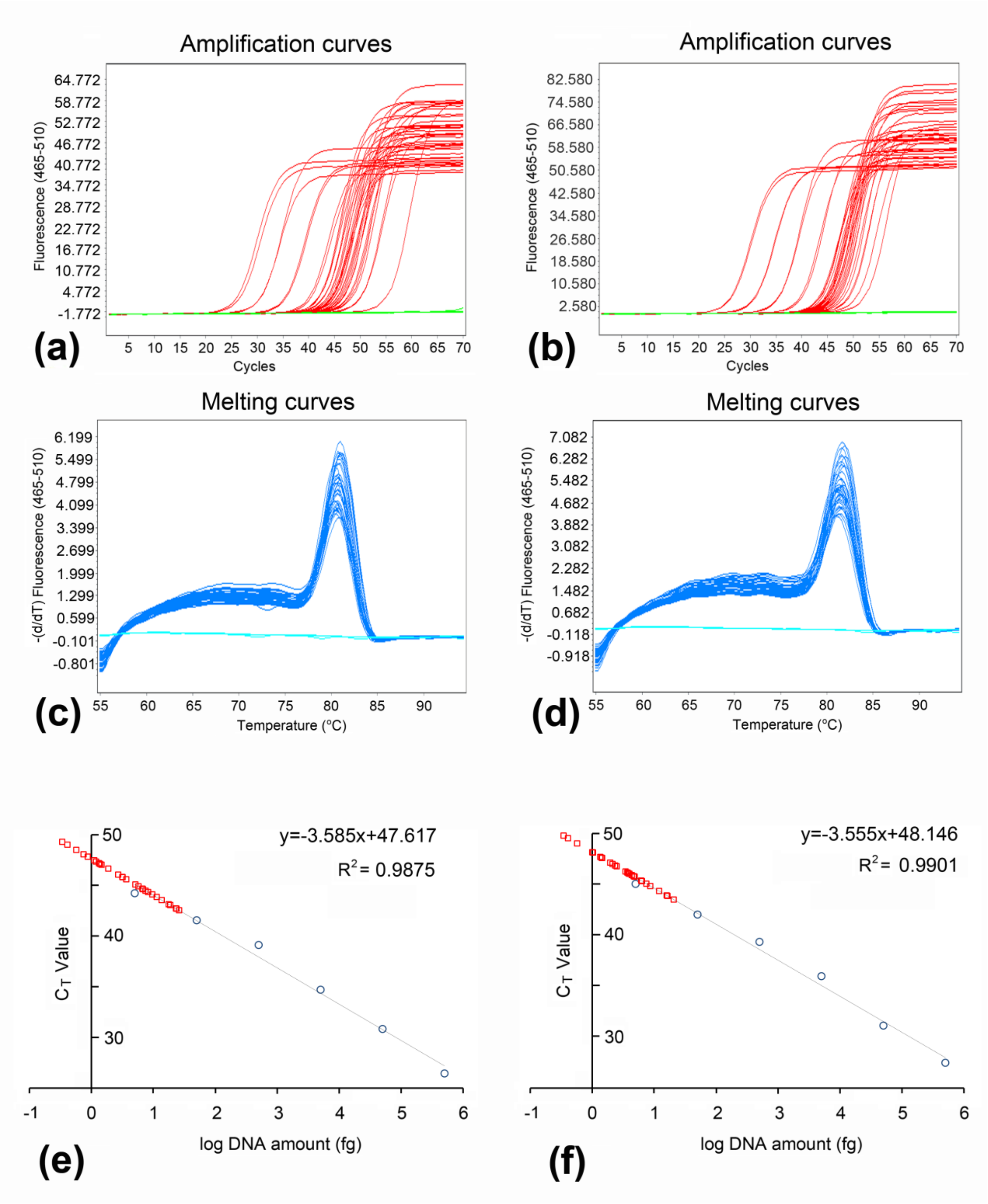

Supplementary Fig. S4 Results of the single-cell qPCR assays. Two of the triplicates were shown here. (a) and (b) were amplification curves of single-cell droplet samples and standard DNA. The x axis represents PCR cycle number and the y axis represents fluorescence intensity. (c) and (d) were melting curves of these samples. The x axis represents melting temperatures and the y axis represents the -ΔF/ΔT (change in fluorescence/change in temperature). (e) and (f) were linear fittings of log transformed DNA concentrations *vs.* C_T values using standard DNA samples (dots). The single-cell droplet samples (squares) were plotted on the fitting curve by C_T values.

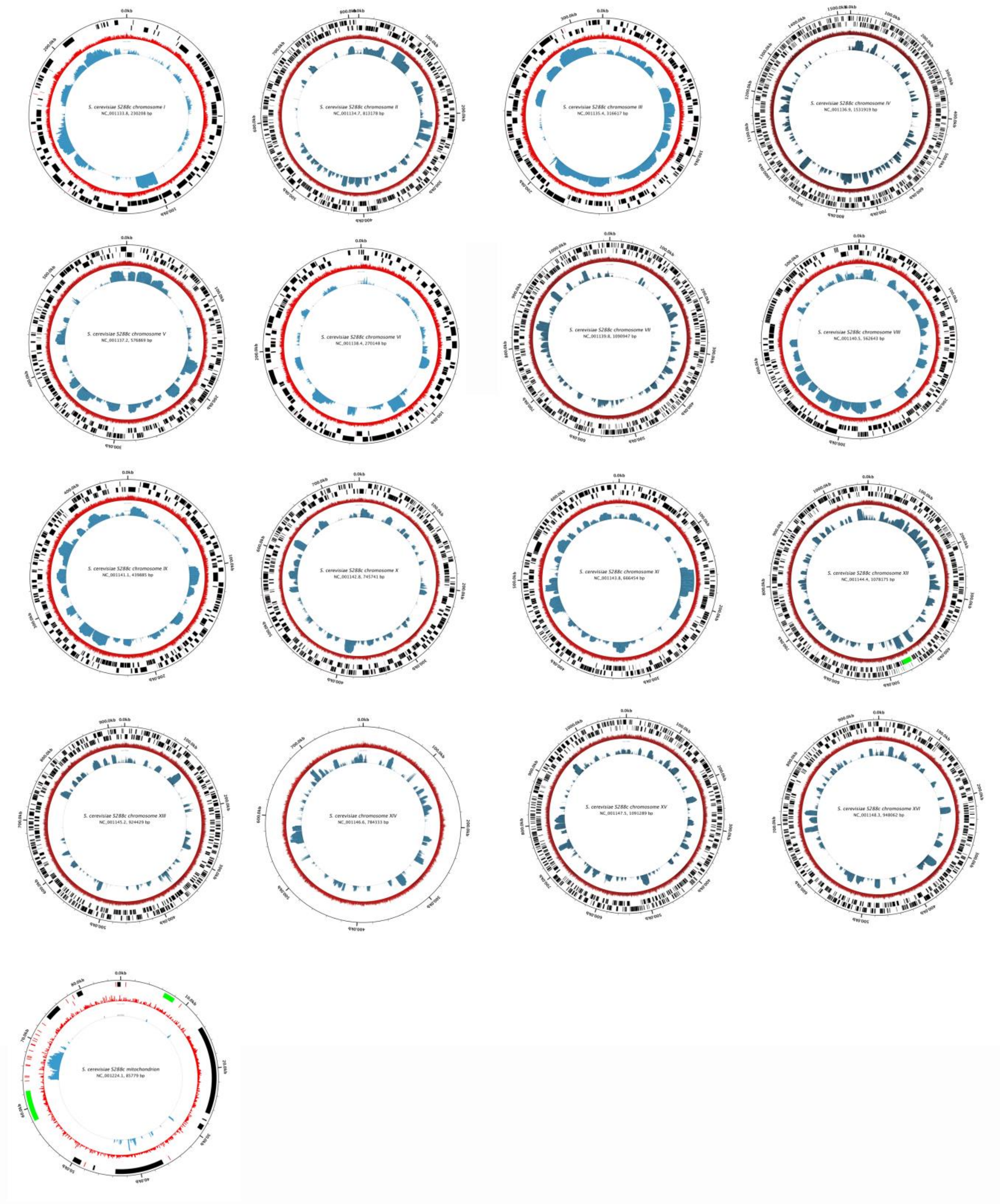

Supplementary Fig. S5 The Circleator figure of reads aligned to the *S. cerevisiae* S288c genome. From outside to inside: coordinate labels of the S288c genome; forward and reverse strand genes of the S288c genome; percent GC content of the sequencing reads of the single-cell sample (shown in red); read coverage of the single-cell sample (shown in blue). The chromosome name, NCBI accession and size were listed. Reads aligned to each of the 16 chromosomes as well as the mitochondrial DNA were shown in individual figures.

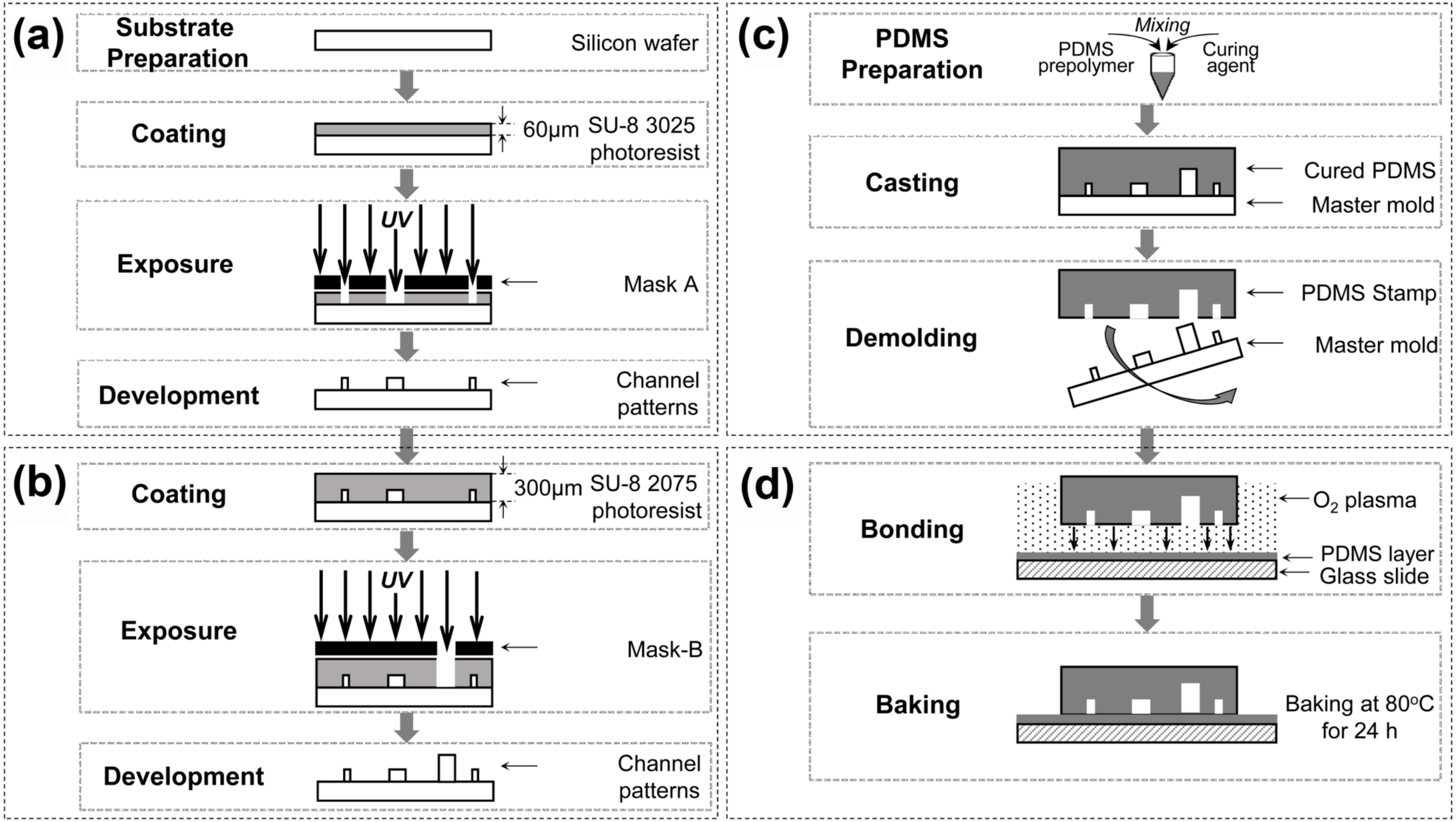

Supplementary Fig. S6 Step-by-step fabrication of the microfluidic chip. (a) Fabrication of the microchannels. (b) Fabrication of the despensing channel. (c) Preparation of the PDMS chip. (d) Assembly of the PDMS chip and the glass substrate.

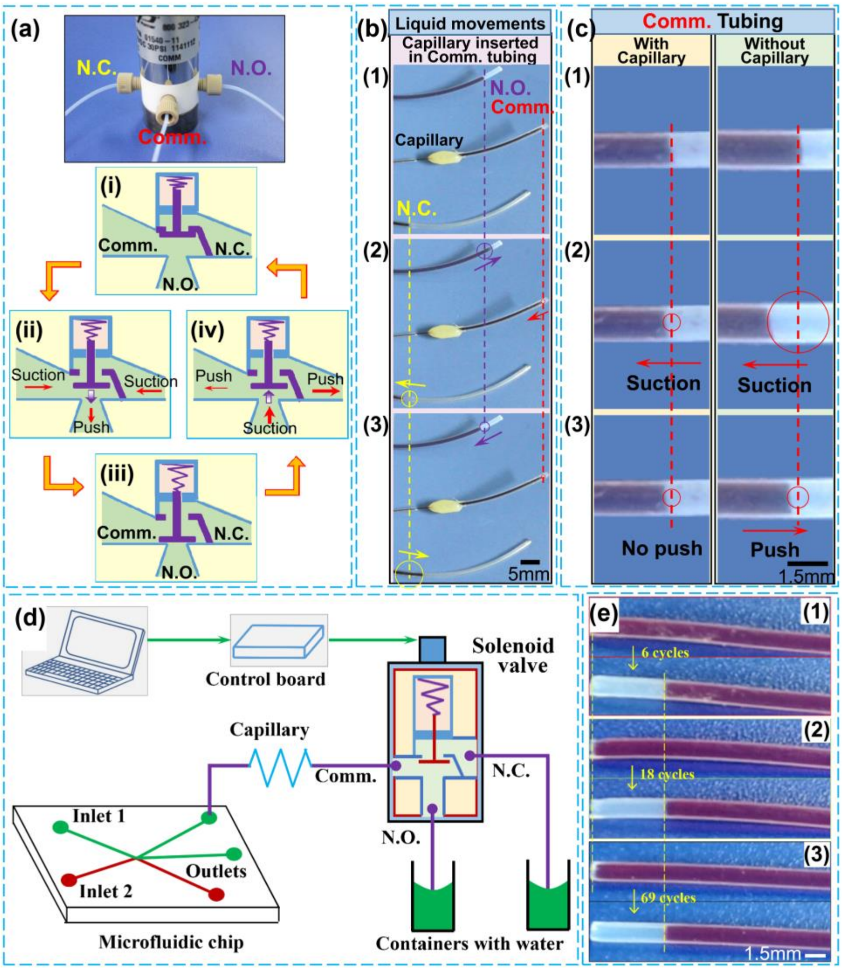

Supplementary Fig. S7 Capillary-tuned solenoid valve suction. (a) Schematics of the operating principle of the solenoid valve in a full work cycle: being energized from (i) to (ii) then to (iii) and being de-energized from (iii) to (iv) and then back to (i). (b) Results of the capillary-tuned solenoid valve suction. Three different colored arrows ("purple" for "N.O.", "red" for "Comm.", and "yellow" for "N.C.") show the directions of the movement of the liquid/air interfaces in each tubing connected with the related port of the solenoid valve while the valve was actuated ("Comm." tubing inserted with a piece of capillary): (1) a de-energized state; (2) the energized state after being energized from (1); (3) the new de-energized state after being de-energized from (2). (c) Two sets of zoomed pictures from (b) showing the liquid/air interfaces in "Comm." tubing, with and without the capillary. (d) The assembly of solenoid-valve-suction based system. (e) The suction volume can be used to indirectly evaluate the suction force. The suction force can be adjusted by changing the hydrodynamic resistance of the inserted capillary. Adapted from Ref. 15 with permission from The Royal Society of Chemistry.

Supplementary Video

Supplementary Video S1 The whole process of single-cell isolation.

References

1. Johnson, E.A. Phaffia rhodozyma: colorful odyssey. *Int. Microbiol.* **6**, 169-174 (2003).
2. Ji, Y. *et al.* Raman spectroscopy provides a rapid, non-invasive method for quantitation of starch in live, unicellular microalgae. *Biotechnol. J.* **9**, 1512-1518 (2014).
3. Teste, M.A. *et al.* Validation of reference genes for quantitative expression analysis by real-time RT-PCR in Saccharomyces cerevisiae. *BMC Mol. Biol.* **10**, 99 (2009).
4. Picelli, S. *et al.* Full-length RNA-seq from single cells using Smart-seq2. *Nat. Protoc.* **9**, 171-181 (2014).
5. Meuser, J.E. *et al.* Phenotypic diversity of hydrogen production in chlorophycean algae reflects distinct anaerobic metabolisms. *J. Biotechnol.* **142**, 21-30 (2009).
6. Dean, F.B. *et al.* Rapid amplification of plasmid and phage DNA using phi29 DNA polymerase and multiply-primed rolling circle amplification. *Genome Res.* **11**, 1095-1099 (2001).
7. Rinke, C. *et al.* Insights into the phylogeny and coding potential of microbial dark matter. *Nature* **499**, 431-437 (2013).
8. Woyke, T. *et al.* Decontamination of MDA reagents for single cell whole genome amplification. *PLoS One* **6**, e26161 (2011).
9. Kurtzman, C.P. & Robnett, C.J. Identification and phylogeny of ascomycetous yeasts from analysis of nuclear large subunit (26S) ribosomal DNA partial sequences. *Antonie Van Leeuwenhoek* **73**, 331-371 (1998).
10. Kim, D.H., Brunt, J. & Austin, B. Microbial diversity of intestinal contents and mucus in rainbow trout (Oncorhynchus mykiss). *J. Appl. Microbiol.* **102**, 1654-1664 (2007).
11. Lasken, R.S. Single-cell genomic sequencing using Multiple Displacement Amplification. *Curr. Opin. Microbiol.* **10**, 510-516 (2007).
12. Langmead, B. *et al.* Ultrafast and memory-efficient alignment of short DNA sequences to the human genome. *Genome Biol.* **10**, R25 (2009).
13. Peng, Y. *et al.* IDBA-UD: a de novo assembler for single-cell and metagenomic sequencing data with highly uneven depth. *Bioinformatics* **28**, 1420-1428 (2012).
14. Crabtree, J. *et al.* Circleator: flexible circular visualization of genome-associated data with BioPerl and SVG. *Bioinformatics* **30**, 3125-3127 (2014).
15. Zhang, Q. *et al.* On-demand control of microfluidic flow via capillary-tuned solenoid microvalve suction. *Lab Chip* **14**, 4599-4603 (2014).
